# Serum apelin-12 and obesity-related markers in Egyptian children with Down syndrome

**DOI:** 10.1007/s00431-023-05315-3

**Published:** 2023-11-06

**Authors:** Sohier Yahia, Nanees A. Salem, Amany El-Hawary, Mohamed Salem, Reham M. El-Farahaty, Abd El-Hady EL-gilany, Rasha M. S. Shoaib, Mohamed Ahmed Noureldin

**Affiliations:** 1 Department of Pediatrics, Genetics Unit, Faculty of Medicine, Mansoura University, Mansoura, Egypt; 2 Department of Pediatrics, Endocrinology Unit, Faculty of Medicine, Mansoura University, Mansoura, Egypt; 3 Mansoura University Children’s Hospital, El-Gomhoria St, Post Office 35516, Box 50, Mansoura, 53355 Egypt; 4 Mansoura General Hospital, Mansoura, Egypt; 5 Clinical Pathology Department, Faculty of Medicine, Mansoura University, Mansoura, Egypt; 6 Public Health Department, Faculty of Medicine, Mansoura University, Mansoura, Egypt; 7 Food and Dairy Sciences and Technology Department, Faculty of Environmental Agricultural Sciences, Arish University, El-Arish, Egypt; 8 Department of Pediatrics, Faculty of Medicine, Horus University, Damietta, Egypt

**Keywords:** Down syndrome, Obesity, Metabolic syndrome, Apelin-12

## Abstract

Children with Down syndrome (DS) exhibit higher overweight/obesity rates than their typically developing peers. Apelin-12 is a bioactive adipokine that exerts vital roles in obesity-related cardiometabolic comorbidities. To date, apelin-12 has not been investigated in obese-DS. This study aimed to explore the possible association between serum apelin-12 and obesity-related markers and to evaluate the efficiency of apelin-12 in the prediction of metabolic syndrome (MetS) in obese-DS compared to BMI Z-score matched obese-control. The cross-sectional study included 150 prepubertal children classified into three groups; obese-DS (*n* = 50), obese-control (*n* = 50), and normal-weight-control (*n* = 50). Anthropometric parameters, body adiposity, fasting serum levels of blood glucose (FBG), insulin, lipid profile, and apelin-12 were evaluated. Homeostasis model assessment of insulin resistance (HOMA-IR) was calculated from FBG and insulin. MetS was defined using Adult Treatment Panel III criteria modified for the pediatric age group. ROC curves were analyzed to evaluate the efficiency of apelin-12 in predicting MetS in obesity groups. Obese-DS exhibited higher body adiposity with marked central fat distribution, atherogenic lipid profile, and higher HOMA-IR compared to obese-control. Apelin-12 was significantly higher in obese-DS and obese-DS with MetS compared to obese-control and obese-control with MetS respectively (*p* < 0.001). The increase in apelin-12 with higher obesity grades was pronounced in obese-DS. Apelin-12 strongly correlated with body adiposity, several MetS risk factors, and HOMA-IR in obese-DS. Significantly higher AUC for apelin-12 in the diagnosis of MetS among obese-DS than obese-control (AUC = 0.948 vs. AUC = 0.807; *p* = 0.04).

*Conclusions*: The current study supports the crucial role of apelin-12 in obesity-related clinical and biochemical markers and in MetS in obese-DS and obese-control. Serum apelin-12 is a potential diagnostic biomarker for MetS with greater performance in obese-DS than obese-control raising its potential for clinical and therapeutic applications.

**Table:** 

**What is Known:** *• Obese-DS children displayed excess body adiposity, Pronounced central fat distribution, atherogenic lipid profile, higher HOMA-IR, and higher prevalence of MetS than obese-control.*	
**What is New:** *• Higher serum apelin-12 was observed in obese-DS and obese-DS with MetS than obese-control and obese-control with MetS respectively. The increase in apelin-12 level with increasing obesity grades was more pronounced in obese-DS.* *• Apelin-12 strongly correlated with obesity-related markers and MetS components in obese-DS. Apelin-12 performed better as a diagnostic biomarker for MetS in obese-DS than obese-control.*

**What is Known:**

*• Obese-DS children displayed excess body adiposity, Pronounced central fat distribution, atherogenic lipid profile, higher HOMA-IR, and higher prevalence of MetS than obese-control.*

**What is New:**

*• Higher serum apelin-12 was observed in obese-DS and obese-DS with MetS than obese-control and obese-control with MetS respectively. The increase in apelin-12 level with increasing obesity grades was more pronounced in obese-DS.*

*• Apelin-12 strongly correlated with obesity-related markers and MetS components in obese-DS. Apelin-12 performed better as a diagnostic biomarker for MetS in obese-DS than obese-control.*

## Introduction

Down syndrome (DS) is the most common chromosomal disorder with an estimated birth prevalence ranging from 6.1 to 13.1/10,000 live births [1]. Fifty percent of children with DS are obese or at risk of becoming obese in their later years [2].

The etiopathogenesis of obesity in DS is multifactorial including, genetic, environmental, social, and most importantly dietary factors [3]. Obesity in children with DS increases the risk of developing metabolic syndrome (MetS) [4]. MetS contribute to the development of adverse cardiometabolic risk in early adulthood [5].

The complexity of obesity and obesity-related comorbidities have become clearer since the discovery of adipokines [6]. Apelin is a new bioactive adipokine involved in many physiological and metabolic processes including fluid balance, food intake, blood pressure regulation, and glucose and lipid metabolism [7, 8]. Apelin synthesis in adipocytes is elicited by insulin and its plasma level is increased in relation to insulin resistance and hyperinsulinemia [9].

The higher risk of overweight and obesity among children with DS versus the general young population has attracted the attention of researchers. We previously explored body adiposity and metabolic parameters in relation to serum adiponectin in a cohort of obese Egyptian children with DS compared to matched obese-controls [10].

Very few studies have investigated apelin-12 levels in obese children with metabolic disorders, but no such studies involving obese children with DS. Therefore, based on the regulatory role of apelin-12 in several metabolic processes, we aimed to investigate for the first time the association between serum apelin-12 level and obesity-related clinical and biochemical parameters and MetS components as well as to explore the efficiency of serum apelin-12 as a diagnostic biomarker for MetS in obese children with DS compared to matched obese-controls. It is worth mentioning that this study was conducted on the same cohort of obese Egyptian children with DS who participated in a previously published study [10].

## Methods

This is a cross-sectional study conducted on the same cohort that participated in our previously published study [10]. The study included 150 prepubertal children (6–11 years) of the same ethnicity categorized into three groups; obese-DS (*n* = 50), obese-control (*n* = 50), and normal-weight-control (*n* = 50). All children with DS had trisomy 21 (nondisjunction).

Children in obese-DS and obese-control groups were recruited between January 2021 and June 2022 from Pediatric Endocrinology and Genetics outpatient clinics at Mansoura University Children’s Hospital. Normal-weight-controls were recruited from a primary school at the same locality. Overall, participants belong to middle-class families and none reported participation in organized physical activity. All participants have normal thyroid profiles.

We excluded children with DS due to translocation/mosaicism, who had secondary obesity, who had major congenital malformations, suffering from chronic comorbidities, or receiving medication known to affect the study variables.

### Clinical evaluation

Body weight and height of participants were measured according to standardized techniques. Body mass index (BMI) was calculated by dividing weight (kg) by height squared (m^2^). Waist circumference (WC) was measured at the midpoint between the lowest ribs and the iliac crests at the end of normal expiration. For all participants, height and BMI *Z*-scores were calculated using reference data for Egyptian children [11]. BMI *Z*-scores in obese children with DS who were short-for-age (height-for-age *Z*-scores <  − 2SD) were adjusted for height-age [13], to avoid the bias in assessing the nutritional status using the absolute BMI values if compared to peers of the same chronological age [12]. Obesity was defined according to the World Health Organization criteria as a BMI *Z*-score above 2SD [14]. Obesity was further classified based on the percentage of BMI above the 95th BMI percentile into three obesity classes; moderate obesity (%BMI ≥ 100%); severe obesity (%BMI ≥ 120%), and morbid obesity (%BMI ≥ 140%) [15].

Blood pressure both systolic and diastolic (SBP/DBP) was obtained using conventional mercury sphygmomanometer following standard technique [16]. Pubertal development was determined in all participants using Tanner classifications. For the purpose of the study, we included prepubertal children only to investigate the study variables independent of the impact of sex steroids.

### Body composition measurements

Body composition measurements were obtained by bioimpedance technique using a Tanita BC-418MA body composition analyzer (Tanita Corp., Tokyo, Japan). We strictly followed the instructions provided by the manufacturer. Body adiposity indicators include the percentage of total body fat (TB-F%), total body-fat mass (TB-FM; kg), trunk-FM (kg), a marker of central adiposity, and appendicular FM (kg) as a sum of the FM in the four limbs that reflect peripheral fat distribution. Trunk-FM to appendicular FM ratio was calculated.

### Biochemical evaluation

Blood samples (5 mL) from each child were collected in the morning, after a 12-h overnight fasting and being centrifuged, and then sera were stored at − 20 °C until analysis. Fasting lipid profile including serum total cholesterol (TC) and triglycerides (TGs) was measured by colorimetric kit (Spinreact, Girona, Spain), and high-density lipoprotein cholesterol (HDL-C) was measured by colorimetric kit (Human Diagnostics, Wiesbaden, Germany). Low-density lipoprotein cholesterol (LDL-C) was estimated using the modified Friedewald formula. Non-HDL-C was calculated by subtracting HDL-C from TC and reflected the cholesterol in all atherogenic lipoprotein particles. Serum apelin-12 was determined by enzyme-linked immunosorbent assay (ELISA) (Human Apelin-12 ELISA Kit, Abbexa, UK). All study participants fasted for 12 h after which venous sampling was done. Serum fasting blood glucose (FBG) was measured by endpoint colorimetric reagents supplied by SPINREACT (S.A./S.A.U Ctra. Santa Coloma, 7 E-17176 SANT ESTEVE DE BAS (GI) SPAIN). Serum insulin was measured by quantitative sandwich ELISA (Human Insulin Kit (ab200011), Abcam, USA). Insulin resistance was assessed by the homeostasis model assessment of insulin resistance (HOMA-IR) as proposed by Matthews et al. [17] using this formula: HOMA-IR = FBG (mmol/L) × fasting insulin (µU/mL)/22.5. A HOMA-IR value above 2.5 was the cut-off point to determine insulin resistance in prepubertal children that corresponds to the 90th percentile of healthy children in previous studies [18].

### Definition of metabolic syndrome

Obese children were classified as having MetS if they had at least three of the following five components according to Adult Treatment Panel III (ATP III) criteria modified for the pediatric age group [19]; (1) central adiposity (WC ≥ 90th percentile for age and gender); (2) FBG ≥ 5.6 mmol/L (100 mg/dL); (3) triglycerides ≥ 1.7 mmol/L (150 mg/dL); (4) HDL-C ≤ 1.03 mmol/L (40 mg/dL); and (5) SBP and/or DBP ≥ 90th percentile for age, gender and height percentile.

### Statistical analysis

Data were processed and analyzed using the IBM SPSS statistics program, version 26.0 (Thousand Oaks, CA, USA). Categorical variables were presented as number and percent (*n*%) and were compared by chi-square (chi^2^) or Fischer’s exact test when appropriate. Quantitative variables were tested for normality using the Kolmogrov-Smirnov test assuming normality at a *P* value of more than 0.05. The variables were non-normally distributed; data were presented as median and interquartile range (IQR). Mann–Whitney *U* test was used to compare medians of two groups and the Kruskal–Wallis test was used to compare medians of three groups followed by a post hoc test (Dunn’s test) to detect pair-wise comparison. Correlations between apelin-12 and MetS risk factors were determined using Spearman’s rank-order correlation. Receiver operator characteristic (ROC) curves were created to evaluate the efficiency of serum apelin-12 as a diagnostic biomarker for MetS in obesity groups. The results were expressed as areas under the curves (AUC), 95% confidence intervals (CI), cut-off values of apelin-12, specificity, sensitivity, and accuracy. MedCalc statistical software version 19.6.1 was used for comparing the AUCs of obesity groups. *P*-value < 0.05 was considered statistically significant.

## Results

The study groups were age- and sex-matched, and obesity groups (obese-DS and obese-control) were matched also for BMI *Z*-score (*p* > 0.05). The results of the analysis of general characteristics, anthropometric measurements, body adiposity indices, and biochemical parameters between the studied groups have been described in detail in our previously published study conducted on the same study population [10]. As expected, obesity groups had significantly higher median values of SBP (*p* = 0.034), BMI *Z*-score, WC, TB-fat%, TB-FM, trunk-FM, trunk/appendicular FM ratio, FBG, fasting insulin, HOMA-IR, TC, TGs, LDL-C, non-HDL-C, and apelin-12 (*p* < 0.001) compared to the normal-weight-control group, while HDL-C was significantly lower in obese-DS compared to normal-weight-control group (*p* < 0.001).

Regarding obesity groups, obese-DS group had significantly higher median values of WC (*p* = 0.027), TB-fat% (*p* = 0.036), TB-FM (*p* = 0.001), trunk-FM (*p* < 0.001), trunk/appendicular FM ratio (*p* < 0.001), fasting insulin (*p* = 0.040), HOMA-IR (*p* = 0.038), TC (*p* < 0.001), TGs (*p* = 0.029), LDL-C (*p* < 0.001), non-HDL-C (*p* < 0.001), and apelin-12 (*p* < 0.001), and significantly lower HDL-C (*p* = 0.001) compared to obese-control (Table 1).

**Table 1:** General, anthropometric, body composition, and biochemical characteristics of the study groups [10]

		**Obese-DS (*n* = 50)**	**Obese-control (*n* = 50)**	**Normal-weight-control (*n* = 50)**	***P*-value**
**Age (years)**		9.0 (8.5–9.9)	8.5 (8.0–10.5)	9.3 (7.0–11.0)	0.073
**Sex**	Male	31 (62%)	30 (60%)	27 (54%)	0.712
	Female	19 (38%)	20 (40%)	23 (46%)	
**Systolic blood pressure (mmHg)**		97.7 (85–115)	95.2 (90–110)	85.6 (70–105)	**0.034**^**bc**^
**Diastolic blood pressure (mmHg)**		66.5 (55 − 70)	62.5 (50–70)	55.7 (40–65)	0.188
**Anthropometric parameters**	Height *Z*-score	− 1.07 (− 1.96–0.32)	0.62 (− 0.62–0.85)	0. 29 (− 0.83–0.14)	**0.001**^**ab**^
	BMI *Z*-score	4.8 (3.7–5.2)	4.5 (3.3–4.8)	0.38 (− 0.58–0.6)	** < 0.001**^**bc**^
	WC (cm)	97.7 (81.5–105)	86.5 (78.0–90)	63.0 (58–67.5)	** < 0.001**^**abc**^
**Body adiposity parameters**	TB-fat %	38.3 (30.2–40.7)	31.9 (28.7–36.4)	18.6 (16.6–20.9)	** < 0.001**^**abc**^
	TB-FM (kg)	21.9 (20.3–27.1)	17.7 (12.8–23.5)	4.9 (4.3–5.8)	** < 0.001**^**abc**^
	Trunk FM (kg)	11.1 (9.7–12.6)	7.9 (5.8–10.9)	1.7 (1.3–2.3)	** < 0.001**^**abc**^
	Appendicular FM (kg)	10.2 (8.9–13.0)	9.0 (7.1–12.9)	3.3 (2.9–4.9)	** < 0.001**^**bc**^
	Trunk/appendicular FM ratio	1.1 (0.96–1.2)	0.82 (0.75–0.91)	0.53 (0.36–0.66)	** < 0.001**^**abc**^
**Biochemical parameters**	FBG (mmol/L)	5.56 (5.11–5.83)	5.44 (5.0–5.67)	4.44 (4.11–4.72)	** < 0.001**^**bc**^
	Fasting insulin (µU/mL)	23.1 (12.8–28.7)	15.5 (7.9–24.9)	6.6 (4.5–7.8)	** < 0.001**^**abc**^
	HOMA-IR	8.7 (3.1–10.2)	5.2 (1.8–9.7)	1.3 (0.96–1.6)	** < 0.001**^**abc**^
	Total Cholesterol (mmol/L)	5.9 (4.8–7.0)	3.9 (3.6–5.2)	3.4 (3.3–3.6)	** < 0.001**^**abc**^
	Triglycerides	1.77 (1.21–1.94)	1.47 (0.87–1.78)	0.89 (0.73–1.07)	** < 0.001**^**abc**^
	LDL (mmol/L)	3.6 (3.1–4.3)	2.9 (2.6–3.3)	2.3 (2.0–2.5)	** < 0.001**^**abc**^
	HDL-C (mmol/L)	1.23 (1.03–1.29)	1.36 (1.16–1.42)	1.32 (1.24–1.68)	**0.001**^**ab**^
	Non-HDL (mmol/L)	5.1 (3.9–6.2)	3.1 (2.6–4.4)	2.3 (2.0–2.5)	** < 0.001**^**abc**^
	Apelin-12 (pg/mL)	850 (600–1712.5)	250 (115–462.5)	75 (65–81.3)	** < 0.001**^**abc**^

All data presented as median and IQR (1st quartile–3rd quartile) except sex presented as frequency number (%)

Bold *P*-value reflects significant difference

*BMI* body mass index, *DS* Down syndrome, *FBG* fasting blood glucose, *HOMA-IR* homeostasis model assessment of insulin resistance, *HDL-C* high-density lipoprotein cholesterol, *LDL-C* low-density lipoprotein cholesterol, non-HDL-C (total cholesterol minus HDL-C), *TB-FM* total body-fat mass, *TB-fat%* total body-fat percentage, *WC* waist circumference

^a^Comparison between obese-DS and obese-control

^b^Comparison between obese-DS and normal-weight-control

^c^Comparison between obese-control and normal-weight-control

**Fig. 1:**
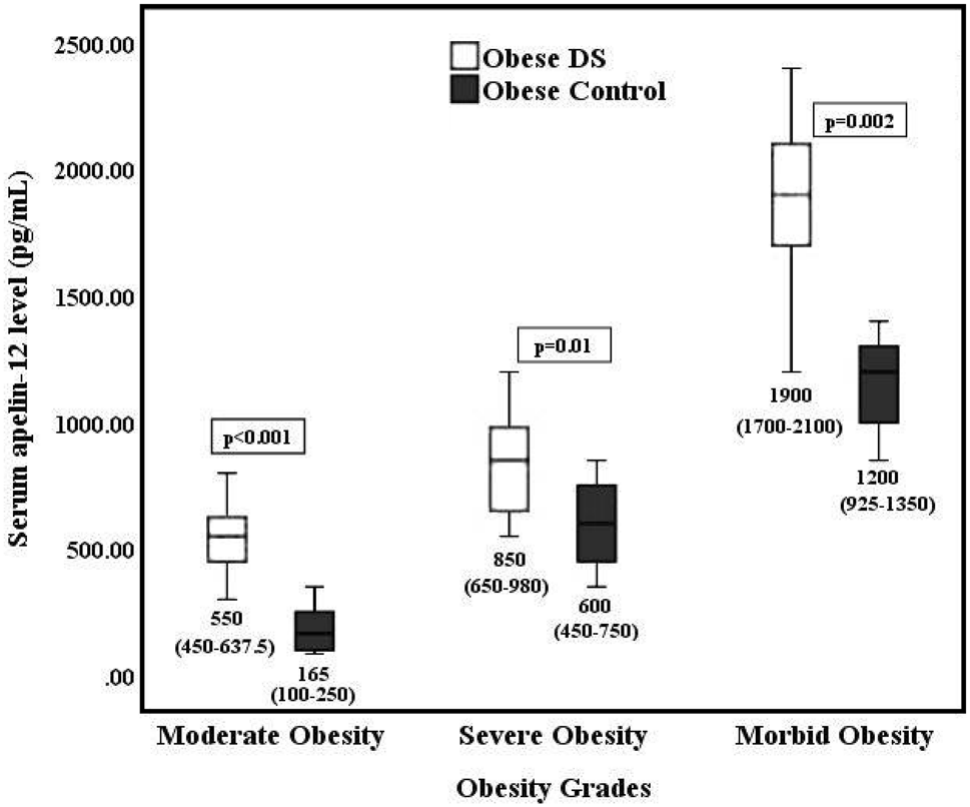
Serum apelin-12 levels among obesity groups according to the obesity grades

Based on the obesity classes, serum apelin-12 levels were significantly elevated in obese-DS compared to obese-control in moderate obesity (*p* < 0.001), severe obesity (*p* = 0.01), and morbid obesity (*p* = 0.002) classes. Within the obese-DS group, serum apelin-12 was significantly higher in severe obesity compared to moderate obesity (*p* = 0.003) and in morbid obesity compared to severe obesity class (*p* < 0.001). Similar results have been detected within obese-control (*p* < 0.001) **(**Fig. 1**)**.

The frequency of MetS was significantly higher in obese-DS (56%) compared to obese-control (34%) (*p* = 0.016). Obese-DS children with MetS were significantly younger in age (*p* = 0.019) and exhibited significantly higher median values of WC (*p* = 0.037), TB-FM (*p* = 0.033), trunk-FM (*p* = 0.004), trunk/appendicular FM ratio (*p* < 0.001), fasting insulin (*p* < 0.001), HOMA-IR (*p* = 0.007), total cholesterol (*p* = 0.012), LDL-C (*p* = 0.003) and non-HDL-C (*p* = 0.007), and apelin-12 (p < 0.001) compared to obese-control with MetS **(**Table 2**)**.

**Table 2:** General, anthropometric, body adiposity, and biochemical characteristics of children who had metabolic syndrome (MetS +) within obesity groups [10]

		**Obese-DS MetS + (*n* = 28)**	**Obese-control MetS + (*n* = 16)**	***P*-value**
**Age (years)**		8.0 (7.0–10.5)	10.0 (9.0–11)	**0.019**
**Systolic blood pressure (mmHg)**		105.2 (95–120)	99.5 (90–120)	0.087
**Diastolic blood pressure (mmHg)**		63.9 (60–85)	65.4 (65–85)	0.787
**Anthropometric parameters**	Height *Z*-score	− 1.03 (− 1.54–0.76)	0.55 (− 0.82–1.18)	**0.045**
	BMI *Z*-score	5.7 (4.5–6.5)	4.8 (4.1–5.2)	0.215
	WC (cm)	99.3 (88.3–105)	92.0 (87–97)	**0.037**
**Body adiposity parameters**	TB-fat %	36.8 (32.1–41.2)	35.5 (26.3–40.5)	0.276
	TB-FM (kg)	24.7 (21.6–28.8)	22.0 (12.8–25.7)	**0.033**
	Trunk FM (kg)	11.8 (10.2–14.7)	9.2 (6.2–11.6)	**0.004**
	Appendicular FM (kg)	12.7 (9.5–13.6)	11.5 (7.3–14.0)	0.232
	Trunk/appendicular FM ratio	0.97 (0.95–1.3)	0.82 (0.75–0.88)	** < 0.001**
**Biochemical parameters**	FBG (mmol/L)	6.67 (5.31–6.0)	5.50 (5.0–5.67)	0.105
	Insulin (µU/mL)	28.9 (23.8–30.0)	20.3 (11.1–26.3)	** < 0.001**
	HOMA-IR	10.8 (6.8–17.8)	7.5 (2.7–9.4)	**0.007**
	Triglycerides (mmol/L)	1.75 (1.35–1.99)	1.52 (0.87–1.84)	**0.035**
	Total cholesterol (mg/dL)	5.9 (4.8–7.3)	4.7 (3.6–5.8)	**0.012**
	LDL-C (mg/dL)	3.8 (3.1–4.4)	3.2 (2.6–3.5)	**0.003**
	HDL-C (mmol/L)	1.21 (1.03–1.29)	1.24 (1.16–1.42)	0.285
	Non-HDL cholesterol	5.1 (4.1–6.4)	3.6 (2.8–5.1)	**0.007**
	Apelin (pg/mL)	1200 (837.5–1925)	550 (225–850)	** < 0.001**

All data presented as median and IQR (1st quartile–3rd quartile)

Bold *P*-value reflects significant difference

*BMI* body mass index, *DS* Down syndrome, *FBG* fasting blood glucose, *HOMA-IR* homeostasis model assessment of insulin resistance, *HDL-C* high-density lipoprotein cholesterol, *LDL-C* low-density lipoprotein cholesterol, *Non-HDL-C* (total cholesterol minus HDL-C), *TB-FM* total body-fat mass, *TB-fat%* total body-fat percentage, *WC* waist circumference

Significant positive correlations were observed between serum apelin-12 level with age, BMI, WC, TB-fat %, TB-FM, trunk-FM, appendicular FM, FBG, fasting insulin level, HOMA-IR, triglycerides, total cholesterol, LDL, and non-HDL in both obesity groups, while serum apelin-12 level showed significant negative correlations with HDL-C in both obesity groups (Table 3).

**Table 3:** Correlation analysis between serum apelin-12 with metabolic syndrome risk factors among obesity groups

	**Obese-DS**		**Obese-control**	
	***R***	***P***	***r***	***P***
**Age (years)**	0.521	** < 0.001**	0.332	**0.022**
**BMI (kg/m**^**2**^**)**	0.923	** < 0.001**	0.851	** < 0.001**
**WC (cm)**	0.484	** < 0.001**	0.343	**0.01**
**TB-fat %**	0.482	** < 0.001**	0.312	**0.03**
**TB-FM (kg)**	0.511	** < 0.001**	0.392	**0.005**
**Trunk-FM**	0.463	** < 0.001**	0.441	**0.001**
**Appendicular FM**	0.562	** < 0.001**	0.724	** < 0.001**
**FBG (mg/dL)**	0.644	** < 0.001**	0.431	**0.002**
**Fasting insulin (µU/ml)**	0.562	** < 0.001**	0.633	** < 0.001**
**HOMA-IR**	0.521	** < 0.001**	0.691	** < 0.001**
**Triglycerides (mmol/L)**	0.452	**0.001**	0.482	** < 0.001**
**Total cholesterol (mmol/L)**	0.564	** < 0.001**	0.621	** < 0.001**
**LDL-C (mmol/L)**	0.432	**0.002**	0.413	**0.003**
**HDL-C (mmol/L)**	-0.421	**0.002**	-0.542	** < 0.001**
**Non-HDL (mmol/L)**	0.431	**0.002**	0.393	**0.005**

*r* Spearman’s rank-order correlation

Bold *P*-value reflects significant difference

*BMI* body mass index, *DS* Down syndrome, *FBG* fasting blood glucose, *HOMA-IR* homeostasis model assessment of insulin resistance, *HDL-C* high-density lipoprotein cholesterol, *LDL-C* low-density lipoprotein cholesterol, *Non-HDL-C* (total cholesterol minus HDL-C), *TB-FM* total body-fat mass, *TB-fat%* total body-fat percentage, *WC* waist circumference

**Fig. 2:**
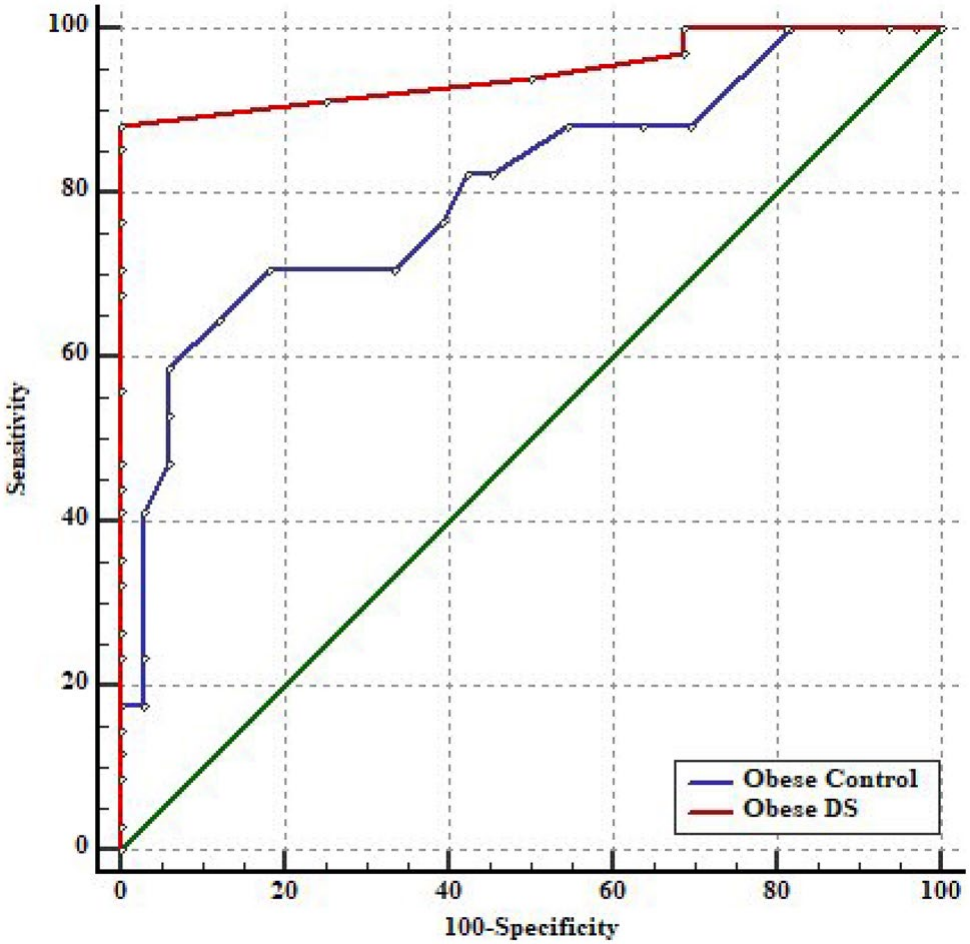
Receiver operating characteristic (ROC) curve for prediction of metabolic syndrome according to serum apelin-12 among obese-DS and obese-control groups

Analysis of The ROC curve analysis was applied to evaluate the power of serum apelin-12 level as a diagnostic marker for MetS revealed that in obese-DS, AUC was 0.948 (95% CI: 0.845–0.991) (*p* < 0.001), and cut-off value was 650 pg/mL with 88.2% sensitivity and 100% specificity and the accuracy was 0.88, while in obese-control, AUC was 0.807 (95% CI: 0.670–0.905) (*p* < 0.001), and cut-off value was 350 pg/mL with 64.71% sensitivity and 90.91% specificity and the accuracy was 0.56. Serum apelin-12 appears to perform better in the prediction of MetS in obese-DS than in obese-control, and there was a significant difference between the two groups in a pairwise comparison of AUCs (*p* = 0.04) **(**Fig. 2**)**.

## Discussion

This study is the first to examine the association between serum apelin-12 and obesity-related clinical and biochemical markers as well as to determine the efficiency of serum apelin-12 in the prediction of MetS in obese-DS compared to matched obese-control.

Although obesity groups were matched for BMI *Z*-score, obese-DS displayed excess body adiposity indicated by TB-fat % and TB-FM with pronounced central fat distribution as shown by higher WC, trunk-FM, and trunk/appendicular FM ratio. Moreover, obese-DS exhibited a prominent atherogenic lipid profile and higher insulin resistance indices (fasting insulin and HOMA-IR values) compared to matched obese-control. Consistent with our findings, body adiposity indicators such as BMI, WC, waist-to-height ratio, %BF, and FM were reported to be significantly higher in obese-DS children and adolescents compared to age- and sex-matched normal-weight-controls [20–22]. All previous observations predispose obese children with DS to MetS.

Practically, the frequency of MetS was significantly higher in obese-DS (68%) compared to matched obese-control (34%). Obese-DS with MetS were significantly younger in age and exhibited significantly higher WC, TB-FM, trunk-FM, trunk/appendicular FM ratio, fasting insulin, HOMA-IR, triglycerides, and non-HDL-C compared to obese-control with MetS.

The high degree of discordance between obese-DS and BMI Z-score matched obese-control in respect to obesity-related clinical and biochemical parameters points to the presence of intrinsic molecular defects, other than traditional risk factors, that could contribute for the development of pathological phenotypes and the high prevalence of metabolic disorders in individuals with DS [23–27].

It has been hypothesized that DS could be a “metabolic disease” [23]. It has been suggested that various genes located on chromosome 21 and involved in the metabolic processes should be overexpressed as a result of the extra copy of this chromosome [23, 24]. Supernumerary copy of the critical region (HSA21) on the long arm of human chromosome 21 has been linked to one-third of the characteristics of DS [25], as well as strongly related to dysregulation of insulin and insulin signaling pathways [23], and has a key role in altered energy metabolism including increased oxidative stress, declined glucose and lipid metabolism, reduced energy production, and mitochondrial dysfunctions [26]. Conversely, some studies have revealed that the genes of the extra copy do not relate perfectly with transcript levels, and other complex molecular pathways are incorporated [28].

Obesity-related comorbidities have been linked to adipokine dysregulation [6]. Apelin-12 is considered one of the more potent forms of apeline involved in many physiological processes and metabolic pathologies [7, 8]. Nowadays, apelin has been demonstrated to assist in regulating glucose and lipid metabolism as it stimulates glucose uptake, increases insulin sensitivity, and regulates lipolysis and fatty acid oxidation [29].

In the current study, we found that apelin-12 was significantly higher in obese groups compared to normal-weight-control, and also was significantly higher in obese-DS compared to matched obese-control. Remarkably, apelin-12 was significantly higher in obese-DS with MetS compared to obese-control with MetS. Based on obesity classes, we identified a significant increase in apelin-12 level with increasing the severity of obesity; this was more prominent in obese-DS than in obese-control. Moreover, serum apelin-12 was significantly correlated to the age, body adiposity indicators, insulin resistance indices, and MetS components in obesity groups. These observations point to the possible crucial role of apelin-12 in the etiopathogenesis of obesity-related clinical and biochemical markers and in the progression from obesity to MetS in obese children with DS.

Studies on the apelin levels in obese adults have shown controversial results. Boucher et al. [30] provided evidence that insulin exerts a direct control on apelin gene expression in adipocytes, and in obese adults, both plasma apelin and insulin levels were significantly higher, suggesting a potential link between apelin and obesity-associated variations of insulin sensitivity status. In addition, Karbek et al. [31] found significantly higher serum apelin levels in patients with MetS than in age-matched controls and significantly associated with coronary atherosclerosis. Moreover, Castan-Laurell et al. [32] observed that a hypocaloric diet associated with weight loss reduces the increased plasma and adipose tissue expression of apelin in obese women. On the other hand, Heinonen et al. [33] reported the absence of significant change in serum apelin in obese adults with MetS who underwent diet-induced weight loss despite reductions in BMI, body adiposity, and mean arterial pressure and enhanced glucose metabolism. The authors suggested that apelin may not be that strongly correlated with FM and it might be involved in the regulation of inflammation and cardiovascular tone.

Very few studies have investigated apelin-12 levels in obese children with metabolic disorders, and no such studies have involved obese children with DS. Our findings are in agreement with a recent study conducted by Yin et al. [34] who reported significantly higher serum apelin-12 in obese children with MetS compared to children without MetS, whereas no difference in serum apelin-12 was observed between obese children without MetS and children from the control group. Also, the authors observed that patients belonging to the highest apelin-12 quartile had a higher prevalence of hypertriglyceridemia, hypo-high-density lipoproteinemia, and MetS when compared with that seen in children belonging to the lowest quartile. Contrarily, Reinehr et al. [35] have reported a lack of association between apelin, insulin resistance, cardiovascular risk factors, and obesity in children. Consistent with its supposed role as an adipokine, high plasma apelin has been indicated by several studies in severe obesity and correlated with body composition [36, 37].

Considerable progress has been made in elucidating the key role of insulin resistance as a candidate link between obesity and obesity-related cardiovascular risk factors. Therefore, the clustering of adverse cardiovascular risk factors together has been named “insulin-resistance syndrome” [38]. Apelin-12 has been related to insulin resistance in recent years. It was investigated that the synthesis of apelin-12 in adipocytes is triggered by insulin, and its serum level is found to be increased in diabetes mellitus, hyperinsulinemia, and insulin resistance [9, 39]. The results of the current study are in agreement with previous studies that revealed the crucial role of apelin-12 in obesity and insulin resistance in children [40, 41]. Previous studies demonstrated that apelin directly enhances insulin sensitivity and suggested that the circulating apelin elevations observed in insulin resistance states are compensatory [42]. Because of these relations, it was suggested that apelin may act as an insulin-sensitizing agent and may be a potential target for the treatment of diabetes, given its effective activity in energy metabolism and insulin sensitivity improvement [43]. In preclinical animal models, insulin sensitivity was improved by direct administration of apelin [36, 44], suggesting its potential for therapeutic applicability.

To determine the efficacy of apelin-12 in the prediction of MetS in both obesity groups, the analysis of ROC curves revealed the greater performance of serum apelin-12 in obese-DS (AUC = 0.948) compared to obese-control (AUC = 0.807), with a higher cut-off value in obese-DS compared to obese-control group (650 pg/mL vs. 350 pg/mL, respectively). Moreover, we observed that apelin-12 is a more sensitive predictor of MetS than adiponectin in obese-DS children based on the results of our published study in which the results of ROC curve analysis for adiponectin in obese-DS children revealed the AUC was 0.808 [10]. In a recent study conducted by Yin et al. [34], the optimal cutoff point for apelin-12 level to predict MetS in obese children was 1.44 ng/mL with AUC = 0.63 and *P* value = 0.046 was reported.

## Study limitations and strengths

There are several limitations in our study including the cross-sectional design, which precludes us from identifying the causal direction between serum apelin-12 and obesity-related clinical and biochemical markers and MetS components in children with DS. In addition to the small sample size that was related to the children being recruited from a single-center and the strict inclusion and exclusion criteria so the results may be preliminary. However, our study is the first to explore serum apelin-12 levels among prepubertal obese-DS children compared to age- and BMI-matched obese-controls. There is a need for further large-scale multicenter longitudinal studies to confirm our findings in this vulnerable population.

## Conclusion

The current study supports the crucial role of apelin-12 in obesity-related clinical and biochemical markers and in MetS in obese-DS and obese-control. Serum apelin-12 efficiently predicts MetS in obese children with greater performance in obese-DS than in matched obese-control raising its potential role for clinical and therapeutic applications in the fields of obesity-related comorbidities and MetS.

## Data Availability

The data sets generated during and/or analyzed during the current study are available from the corresponding author on reasonable request.

## Abbreviations

ATP III: Adult Treatment Panel III
DBP: Diastolic BP
DS: Down syndrome
HDL-C: High-density lipoprotein cholesterol
HOMA-IR: Homeostasis model assessment of insulin resistance
HSA21: Human chromosome 21
LDL-C: Low-density lipoprotein cholesterol
MetS: Metabolic syndrome
Non-HDL-C: Non-high-density lipoprotein cholesterol
SBP: Systolic BP
TC: Total cholesterol
TGs: Triglycerides
WC: Waist circumference

## References

[1] Presson AP, Partyka G, Jensen KM, Devine OJ, Rasmussen SA, McCabe LL, McCabe ER (2013). Current estimate of Down syndrome population prevalence in the United States. J Pediatr.

[2] Martínez-Espinosa RM, Molina Vila MD, Reig García-Galbis M (2020) Evidences from clinical trials in Down syndrome: diet, exercise and body composition. Int J Environ Res Public Health 16;17(12):4294. 10.3390/ijerph17124294 10.3390/ijerph17124294 PMC7344556 32560141

[3] Sahoo K, Sahoo B, Choudhury AK, Sofi NY, Kumar R, Bhadoria AS (2015). Childhood obesity: causes and consequences. J Family Med Prim Care.

[4] Bertapelli F, Pitetti K, Agiovlasitis S, Guerra-Junior G (2016). Overweight and obesity in children and adolescents with Down syndrome-prevalence, determinants, consequences, and interventions: a literature review. Res Dev Disabil.

[5] Yajnik CS, Katre PA, Joshi SM, Kumaran K, Bhat DS, Lubree HG, Memane N, Kinare AS, Pandit AN, Bhave SA, Bavdekar A, Fall CH (2015). Higher glucose, insulin and insulin resistance [HOMA-IR] in childhood predict adverse cardiovascular risk in early adulthood: the Pune Children’s study. Diabetologia.

[6] Weschenfelder C, Schaan de Quadros A, Lorenzon Dos Santos J, Bueno Garofallo S, Marcadenti A (2020) Adipokines and adipose tissue-related metabolites, nuts and cardiovascular disease. Metabolites 11;10(1):32. 10.3390/metabo10010032 10.3390/metabo10010032 PMC7022531 31940832

[7] Schinzari F, Veneziani A, Mores N, Barini A, Di Daniele N, Cardillo C, Tesauro M (2017). Beneficial effects of apelin on vascular function in patients with central obesity. Hypertension.

[8] Hu G, Wang Z, Zhang R, Sun W, Chen X (2021). The role of apelin/apelin receptor in energy metabolism and water homeostasis: a comprehensive narrative review. Front Physiol.

[9] Jiang Y, Yan M, Wang C, Wang Q, Chen X, Zhang R, Wan L, Ji B, Dong B, Wang H, Chen J (2021). The effects of apelin and elabela ligands on apelin receptor distinct signaling profiles. Front Pharmacol.

[10] Yahia S, El-Farahaty R, El-Gilany AH, Shoaib R, Ramadan R, Salem N (2021) Serum adiponectin, body adiposity and metabolic parameters in obese Egyptian children with Down syndrome. J Pediatr Endocrinol Metab 34(11):1401–1410. 10.1515/jpem-2021-0324 10.1515/jpem-2021-0324 34348423

[11] Ghalli I, Salah N, Hussien F, Erfan M, El- Ruby M, Mazen I, Sabry M, Abd El-am knack Razik M, Saad M, Hossney L, Ismaail S, Abd El-Dayem S (2008) Egyptian growth curves for infants, children and adolescents 2002. Published aka Sartorio A, Buckler JMH and Marazzi N (eds). Crescere nel mondo 2008. Ferring publisher, Italy

[12] Artioli TO, Witsmiszyn E, Belo Ferreira A, Franchi Pinto C (2017). Assessing Down syndrome BMI and body composition. Int Med Rev Down Syndrome.

[13] Bonthuis M, Jager KJ, Abu-Hanna A, Verrina E, Schaefer F, van Stralen KJ (2013). Application of body mass index according to height-age in short and tall children. PLoS ONE.

[14] de Onis M, Onyango AW, Borghi E, Siyam A, Nishida C, Siekmann J (2007). Development of a WHO growth reference for school-aged children and adolescents. Bull World Health Organ.

[15] Skinner AC, Perrin EM, Moss LA, Skelton JA (2015). Cardiometabolic risks and severity of obesity in children and young adults. N Engl J Med.

[16] Flynn JT, Kaelber DC, Baker-Smith CM, Blowey D, Carroll AE, Daniels SR, de Ferranti SD, Dionne JM, Falkner B, Flinn SK, Gidding SS, Goodwin C, Leu MG, Powers ME, Rea C, Samuels J, Simasek M, Thaker VV, Urbina EM (2017) Subcommittee on screening and management of high blood pressure in children. Clinical practice guideline for screening and management of high blood pressure in children and adolescents. Pediatrics 140(3):e20171904. 10.1542/peds.2017-1904 10.1542/peds.2017-1904 28827377

[17] Matthews DR, Hosker JP, Rudenski AS, Naylor BA, Treacher DF, Turner RC (1985). Homeostasis model assessment: insulin resistance and beta-cell function from fasting plasma glucose and insulin concentrations in man. Diabetologia.

[18] Valerio G, Licenziati MR, Iannuzzi A, Franzese A, Siani P, Riccardi G, Rubba P (2006). Insulin resistance and impaired glucose tolerance in obese children and adolescents from Southern Italy. Nutr Metab Cardiovasc Dis.

[19] Steinberger J, Daniels SR, Eckel RH, Hayman L, Lustig RH, McCrindle B, Mietus-Snyder ML (2009) American Heart Association Atherosclerosis, Hypertension, and Obesity in the Young Committee of the Council on Cardiovascular Disease in the Young; Council on Cardiovascular Nursing; and Council on Nutrition, Physical Activity, and Metabolism. Progress and challenges in metabolic syndrome in children and adolescents: a scientific statement from the American Heart Association Atherosclerosis, Hypertension, and Obesity in the Young Committee of the Council on Cardiovascular Disease in the Young; Council on Cardiovascular Nursing; and Council on Nutrition, Physical Activity, and Metabolism. Circulation 119(4):628–47. 10.1161/CIRCULATIONAHA.108.191394 10.1161/CIRCULATIONAHA.108.191394 19139390

[20] González-Agüero A, Vicente-Rodríguez G, Ara I, Moreno LA, Casajús JA (2011). Accuracy of prediction equations to assess percentage of body fat in children and adolescents with Down syndrome compared to air displacement plethysmography. Res Dev Disabil.

[21] Gutierrez-Hervas A, Gómez-Martínez S, Izquierdo-Gómez R, Veiga OL, Perez-Bey A, Castro-Piñero J, Marcos A (2020). Inflammation and fatness in adolescents with and without Down syndrome: UP & DOWN study. J Intellect Disabil Res.

[22] Loveday SJ, Thompson JM, Mitchell EA (2012). Bioelectrical impedance for measuring percentage body fat in young persons with Down syndrome: validation with dual-energy absorptiometry. Acta Paediatr.

[23] Dierssen M, Fructuoso M, Martínez de Lagrán M, Perluigi M, Barone E (2020) Down syndrome is a metabolic disease: altered insulin signaling mediates peripheral and brain dysfunctions. Front Neurosci 14:670. 10.3389/fnins.2020.00670 10.3389/fnins.2020.00670 PMC7360727 32733190

[24] Campos C, Casado Á (2015). Oxidative stress, thyroid dysfunction & Down syndrome. Indian J Med Res.

[25] Pelleri MC, Cicchini E, Locatelli C, Vitale L, Caracausi M, Piovesan A, Rocca A, Poletti G, Seri M, Strippoli P, Cocchi G (2016) Systematic reanalysis of partial trisomy 21 cases with or without Down syndrome suggests a small region on 21q22.13 as critical to the phenotype. Hum Mol Genet 25(12):2525–2538. 10.1093/hmg/ddw116 10.1093/hmg/ddw116 PMC5181629 27106104

[26] Moreau M, Benhaddou S, Dard R, Tolu S, Hamzé R, Vialard F, Movassat J, Janel N (2021). Metabolic diseases and Down syndrome: how are they linked together?. Biomedicines.

[27] Izzo A, Mollo N, Nitti M, Paladino S, Calì G, Genesio R, Bonfiglio F, Cicatiello R, Barbato M, Sarnataro V, Conti A, Nitsch L (2018). Mitochondrial dysfunction in down syndrome: molecular mechanisms and therapeutic targets. Mol Med.

[28] Lana-Elola E, Watson-Scales SD, Fisher EM, Tybulewicz VL (2011). Down syndrome: searching for the genetic culprits. Dis Model Mech.

[29] Li C, Cheng H, Adhikari BK, Wang S, Yang N, Liu W, Sun J, Wang Y (2022). The role of apelin-APJ system in diabetes and obesity. Front Endocrinol (Lausanne).

[30] Boucher J, Masri B, Daviaud D, Gesta S, Guigné C, Mazzucotelli A, Castan-Laurell I, Tack I, Knibiehler B, Carpéné C, Audigier Y, Saulnier-Blache JS, Valet P (2005). Apelin, a newly identified adipokine up-regulated by insulin and obesity. Endocrinology.

[31] Karbek B, Bozkurt NC, Topaloglu O, Aslan MS, Gungunes A, Cakal E, Delibasi T (2014). Relationship of vaspin and apelin levels with insulin resistance and atherosclerosis in metabolic syndrome. Minerva Endocrinol.

[32] Castan-Laurell I, Vítkova M, Daviaud D, Dray C, Kováciková M, Kovacova Z, Hejnova J, Stich V, Valet P (2008). Effect of hypocaloric diet-induced weight loss in obese women on plasma apelin and adipose tissue expression of apelin and APJ. Eur J Endocrinol.

[33] Heinonen MV, Laaksonen DE, Karhu T, Karhunen L, Laitinen T, Kainulainen S, Rissanen A, Niskanen L, Herzig KH (2009). Effect of diet-induced weight loss on plasma apelin and cytokine levels in individuals with the metabolic syndrome. Nutr Metab Cardiovasc Dis.

[34] Yin C, Zhang H, Zhang M, Xiao Y (2020). Adropin and apelin-12 efficiently predict metabolic syndrome in obese children. Pediatr Diabetes.

[35] Reinehr T, Woelfle J, Roth CL (2011). Lack of association between apelin, insulin resistance, cardiovascular risk factors, and obesity in children: a longitudinal analysis. Metabolism.

[36] Higuchi K, Masaki T, Gotoh K, Chiba S, Katsuragi I, Tanaka K, Kakuma T, Yoshimatsu H (2007). Apelin, an APJ receptor ligand, regulates body adiposity and favors the messenger ribonucleic acid expression of uncoupling proteins in mice. Endocrinology.

[37] Frier BC, Williams DB, Wright DC (2009). The effects of apelin treatment on skeletal muscle mitochondrial content. Am J Physiol Regul Integr Comp Physiol.

[38] Caprio S, Santoro N, Weiss R (2020). Childhood obesity and the associated rise in cardiometabolic complications. Nat Metab.

[39] Kolan Ska-Dams E, Boinska J, Socha MW (2021) Adipokine levels and carbohydrate metabolism in patients diagnosed de novo with polycystic ovary syndrome. Qatar Med J 2021(2):34. 10.5339/qmj.2021.34 10.5339/qmj.2021.34 PMC8474077 34604014

[40] Ba HJ, Chen HS, Su Z, Du ML, Chen QL, Li YH, Ma HM (2014) Associations between serum apelin-12 levels and obesity-related markers in Chinese children. PLoS One 9(1):e86577. 10.1371/journal.pone.0086577 10.1371/journal.pone.0086577 PMC3903556 24475149

[41] El Wakeel MA, El-Kassas GM, Kamhawy AH, Galal EM, Nassar MS, Hammad EM, El-Zayat SR (2018) Serum apelin and obesity-related complications in Egyptian children. Open Access Maced J Med Sci 6(8):1354–1358. 10.3889/oamjms.2018.312 10.3889/oamjms.2018.312 PMC6108807 30159056

[42] Castan-Laurell I, Dray C, Knauf C, Kunduzova O, Valet P (2012). Apelin, a promising target for type 2 diabetes treatment?. Trends Endocrinol Metab.

[43] Xu S, Tsao PS, Yue P (2011). Apelin and insulin resistance: another arrow for the quiver?. J Diabetes.

[44] Dray C, Knauf C, Daviaud D, Waget A, Boucher J, Buléon M, Cani PD, Attané C, Guigné C, Carpéné C, Burcelin R, Castan-Laurell I, Valet P (2008). Apelin stimulates glucose utilization in normal and obese insulin-resistant mice. Cell Metab.

